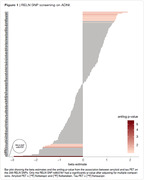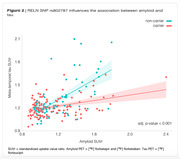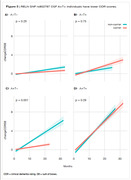# A polymorphism in RELN protects against amyloid‐driven tau pathology and cognitive decline in sporadic Alzheimer’s disease

**DOI:** 10.1002/alz.094034

**Published:** 2025-01-09

**Authors:** Giovanna Carello‐Collar, Thomas Hugentobler Schlickmann, João Pedro Ferrari‐Souza, Marco Antônio de Bastiani, Alexandre Santos Cristino, Tharick A. Pascoal, Pedro Rosa‐Neto, Diogo O. Souza, Eduardo R. Zimmer

**Affiliations:** ^1^ Universidade Federal do Rio Grande do Sul, Porto Alegre, RS Brazil; ^2^ Universidade Federal do Rio Grande do Sul, Porto Alegre, Rio Grande do Sul Brazil; ^3^ Federal University of Rio Grande do Sul, Porto Alegre, Rio Grande do Sul Brazil; ^4^ Griffith Institute for Drug Discovery, Griffith University, Brisbane Australia; ^5^ University of Pittsburgh School of Medicine, Pittsburgh, PA USA; ^6^ Translational Neuroimaging Laboratory, The McGill University Research Centre for Studies in Aging, Montréal, QC Canada; ^7^ Universidade Federal do Rio Grande do Sul, Porto Alegre Brazil

## Abstract

**Background:**

A rare reelin gene variant (RELN‐COLBOS mutation) delayed dementia onset in almost 30 years in an autosomal dominant Alzheimer’s disease (ADAD) carrier. This patient presented with high amyloid‐ß (Aß) plaque load, but low tau accumulation, suggesting that this single‐nucleotide polymorphism (SNP) in RELN conferred a resilience not only to cognitive decline but also to tauopathy in ADAD. However, whether RELN SNPs are also protective in sporadic Alzheimer’s disease (AD) is yet to be determined. Thus, we sought to examine the impact of RELN SNPs on AD pathophysiology and cognitive deterioration in sporadic AD.

**Method:**

We assessed 198 individuals [105 cognitively unimpaired (CU) and 85 cognitively impaired (CI)] from the Alzheimer’s Disease Neuroimaging Initiative (ADNI) with available data on RELN SNPs and amyloid‐ and tau‐PET measures ([18F]‐florbetaben/florbetapir and [18F]‐flortaucipir, respectively), Aß1‐42 and ptau181 in the CSF, and neuropsychological testing (Clinical Dementia Rating Sum of Boxes). We analyzed the effect of RELN SNPs carriership in the association between amyloid and tau burden through linear regression analysis and on cognitive decline according to CSF AT status through linear mixed‐effect model correcting for age, sex, and ApoEe4 status (Bonferroni’s adjusted p‐value < 0.05).

**Result:**

We performed linear regression analysis in all the 235 RELN SNPs available on ADNI (Fig.1). We found RELN rs802787 protected against amyloid‐driven tau pathology (adj. p‐value < 0.001, Fig.2). Dividing individuals according to the CSF AT status, we observed that RELN rs802787 did not impact the rate of decline in cognition in A‐T‐ and A‐T+ individuals (Fig.3A‐B). By contrast, RELN rs802787 CSF A+T‐ individuals presented a slower cognitive decline (Fig.3C), which was not observed in A+T+ individuals (Fig.3D).

**Conclusion:**

Here, we show RELN rs802787 carriers presenting high amyloid load have lower tau accumulation than non‐carriers. In addition, CSF A+T‐ RELN carriers presented a slower cognitive decline compared to non‐carriers. Our results suggest that RELN rs802787 carriership protects against amyloid‐driven tau pathology and cognitive deterioration in sporadic AD individuals. To the best of our knowledge, this is the first RELN SNP found to be protective against non‐familial AD.